# Construction of Customized Palatal Orthodontic Devices on Skeletal Anchorage Using Biomechanical Modeling

**DOI:** 10.3390/bioengineering9010012

**Published:** 2022-01-01

**Authors:** Dmitriy Suetenkov, Dmitriy Ivanov, Aleksandr Dol, Ekaterina Diachkova, Yuriy Vasil’ev, Leonid Kossovich

**Affiliations:** 1 Department of Pediatric Dentistry and Orthodontics, Saratov State Medical University, Bolshaya Kazachya, 112, 410012 Saratov, Russia; suetenkov@gmail.com; 2 Department of Mathematical Theory of Elasticity and Biomechanics, Saratov State University, Astrakhanskaya, 83, 410012 Saratov, Russia; ivanovdv@gmail.com (D.I.); nerevishl@gmail.com (A.D.); president@sgu.ru (L.K.); 3 Department of Oral Surgery of Borovsky Institute of Dentistry, I.M. Sechenov First Moscow State Medical University (Sechenov University), Trubetskaya St. bldg. 8\2, 119991 Moscow, Russia; 4 Department of Operative Surgery and Topographic Anatomy, I.M. Sechenov First Moscow State Medical University (Sechenov University), Trubetskaya St. bldg. 8\2, 119991 Moscow, Russia; y_vasiliev@list.ru

**Keywords:** biomechanical modeling, palatal anchorage devices, mini-implants, orthodontic treatment

## Abstract

Orthodontic implants have been developed for the implementation of skeletal anchorage and are effectively used in the design of individual orthodontic devices. However, despite a significant amount of clinical research, the biomechanical aspects of the use of skeletal anchorage have not been adequately studied. The aim of this work was to numerically investigate the stress–strain state of the developed palatal orthodontic device supported by mini-implants. Four possible options for the placement of mini-implants in the bone were analyzed. The effect of a chewing load of 100 N on the bite plane was investigated. The study was carried out using biomechanical modeling based on the finite element method. The installation of the palatal orthodontic device fixed on mini-implants with an individual bite plane positioned on was simulated. The dependence of equivalent stresses and deformation changes on the number and location of the supporting mini-implants of the palatal orthodontic device was investigated. Two materials (titanium alloy and stainless steel) of the palatal orthodontic device were also investigated. The choice of a successful treatment option was based on the developed biomechanical criteria for assessing the surgical treatment success. Application of the criteria made it possible to estimate the stability and strength of fixation of each of the considered mini-implants installation options. As a result, options for the mini-implants optimal placement were identified (the first and the fourth which provide distributed front and side support of the device), as well as the preferred material (titanium alloy) for the manufacture of the palatal orthodontic device.

## 1. Introduction

Orthodontic implants have been developed to provide skeletal support and are effectively used in the design of individualized orthodontic devices. However, despite a significant amount of clinical research, the biomechanical aspects of the use of some types of skeletal support have not been adequately studied.

Support stability plays an important role in orthodontic treatment and can maximize desired tooth movement as well as minimize unwanted side effects [1]. Orthodontic forces can be applied to both a single implant and support systems, such as palatal support structures or palatal orthodontic devices (POD) [2]. POD has several advantages, such as a simpler surgical protocol, less trauma to the surrounding tissues during insertion [3] and removal, minimal anatomical restrictions, and the possibility of immediate loading after implantation [4]. At the same time, there is evidence of a lower rate of loss of tandem mini-implants compared to single ones, which allows one to speak about the prospects for using POD on several supports [5]. The literature describes two techniques for using skeletal support: direct and indirect support. In this work, an attempt was made to biomechanically substantiate a new effect when using POD—the perception of the occlusal load on the support system of the bite plane. This rationale is necessary for the use of treatment methods previously used on removable orthodontic appliances. The use of such devices requires a high level of patient compliance and creates great problems for eating during a long orthodontic treatment.

Previous studies [6,7,8,9,10,11,12] have assessed the stability of mini-implants and their systems under changing mechanical conditions, the geometry of supporting devices, jawbone tissue properties, implantation conditions, and force effects. The conditions for maintaining the stability of mini-implants, for example, with different thread shapes, the use of screw implants with different diameters and lengths, the value of bone density, the use of implants without or with partial osseointegration were studied.

Due to the peculiarities of POD and attempts to create bite plains of various sizes on them, the transfer of forces and their distribution over the structures of the upper jaw are of known interest. Biomechanical factors can significantly determine the use of this technique in a clinical setting. No such data has been found in the studies known to us. Without a deep understanding of the biomechanical rationale for orthodontic treatment, it is impossible to develop a clear methodology for the clinical use of our proposed therapy method.

The aim of this work was to numerically investigate the stress–strain state of the developed palatal orthodontic device supported by mini-implants under chewing load. The dependence of stress and deformation changes on the number and location of the supporting mini-implants of the planned structure was shown. As a result, options for the mini-implants optimal placement were identified, as well as the preferred material for the manufacture of the palatal orthodontic device.

## 2. Materials and Methods

**Figure 1:**
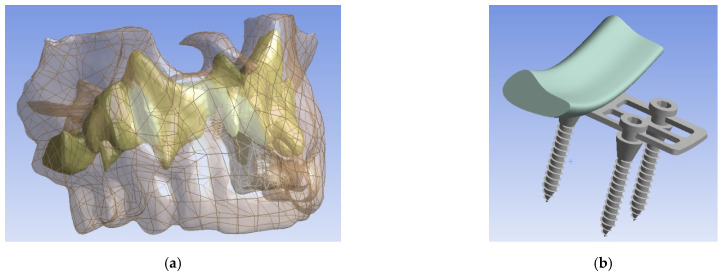
3D solid geometrical models: (**a**) two types of jawbone tissue, transparent—cortical bone, yellow—trabecular bone; (**b**) implant of the first configuration (Model 1).

Numerical modeling of the bone-implant system (upper jaw, mini-implants, bite plate, and POD) stress–strain state was carried out. The human upper jaw was examined. The upper jaw was represented by two tissues (trabecular bone tissue and cortical bone tissue), each of which was modeled as a linearly elastic isotropic and homogeneous body (Figure 1). Trabecular bone young modulus was obtained on the basis of computed tomography data of this patient using an original technique [13].

**Figure 2:**
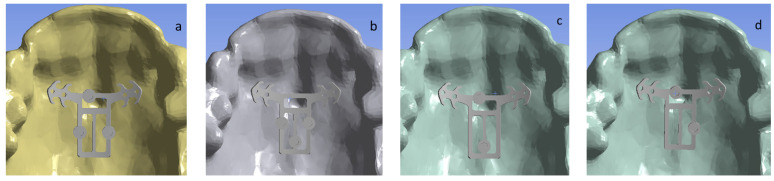
Four investigated mini-implant configurations. Bottom view. (**a**) Model 1. (**b**) Model 2. (**c**) Model 3. (**d**) Model 4.

The upper jaw 3D surface model was built in Mimics software based on CT data. The solid model of the jaw was built using 3Matic software. In the SolidWorks CAD system, 3D models of POD, mini-implants and bite planes were built. Solid implant and jaw models were merged in SolidWorks. Four possible mini-implant configurations were numerically investigated (Figure 2).

**Figure 3:**
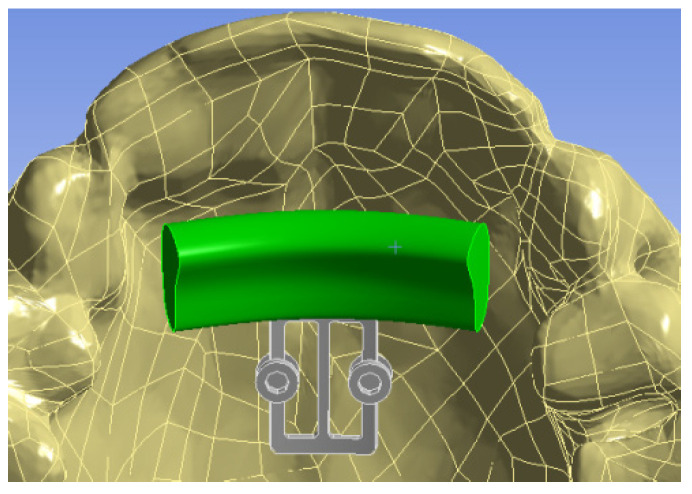
3D jaw model with installed POD and bite plane.

Figure 3 shows the 3D model with the bite plane which is highlighted with green color.

**Figure 4:**
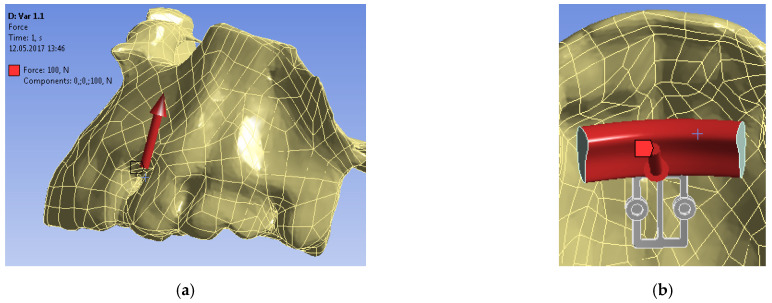
Applied load of 100 N: (**a**) force magnitude and direction; (**b**) force application area.

For each mini-implant configuration, a static theory of elasticity problem was considered—the upper part of the jaw was rigidly fixed, and a constant force of 100 N was applied to the surface of the bite plane (Figure 4) [14,15,16]. Thus, the task of applying a chewing load to the POD was simulated.

The static theory of elasticity problems [17] about the stress–strain state of the bone-implant system was solved numerically using the finite element method in Ansys software. Total displacements and equivalent stresses of the jawbone, mini-implants, and POD under the applied load were analyzed.

**Figure 5:**
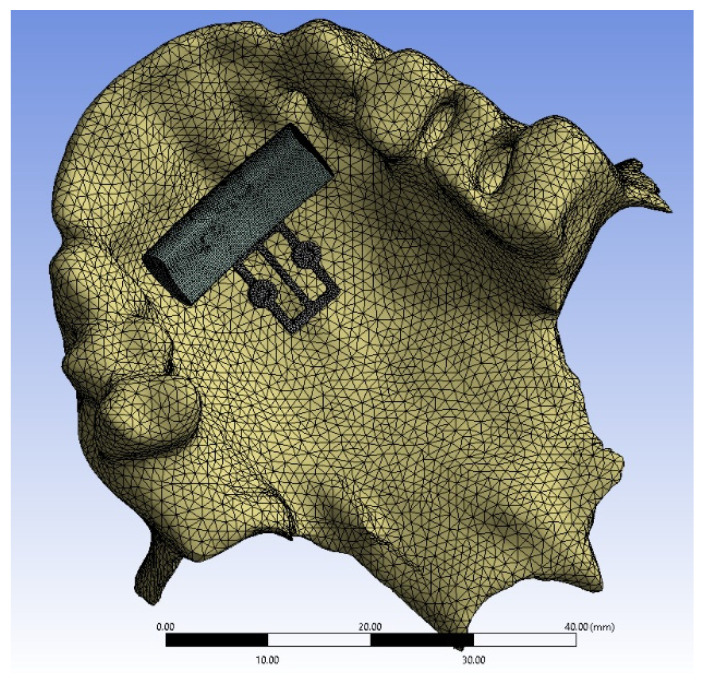
Hybrid computational mesh for the first mini-implant configuration.

Mesh convergence analysis was carried out in Ansys software, which made it possible to determine the size of the mesh element, which had little effect on the numerical results. As a result, for each model under investigation, the number of finite elements was on average 600,000 (Figure 5).

Full contact (bonded contact) conditions were set between POD and mini-implants, as well as between bone and mini-implants [17].

The dependence of the bone-implant system stress–strain state on the type of POD material was analyzed. POD made of stainless steel and titanium alloy was examined. Mini-implants were made of titanium alloy. Mechanical properties of bone tissues, POD, and mini-implants are shown in Table 1.

## 3. Results

### 3.1. Titanium Alloy

**Figure 6:**
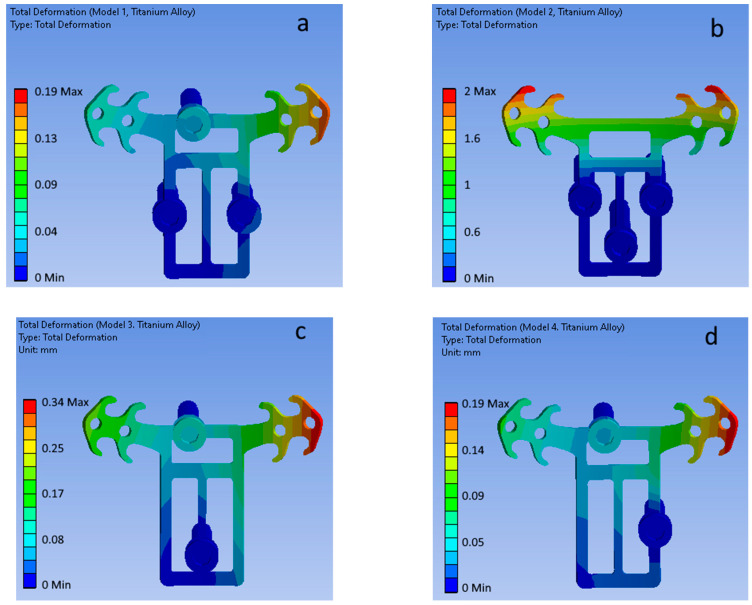
Total displacement fields for (**a**–**d**) mini-implant configurations (POD material—titanium alloy). (**a**) Model 1. (**b**) Model 2. (**c**) Model 3. (**d**) Model 4.

Figure 6 shows the total displacement fields of the POD (Ti6Al4V ELI titanium alloy material) and mini-implants of the four considered configurations (top view).

**Figure 7:**
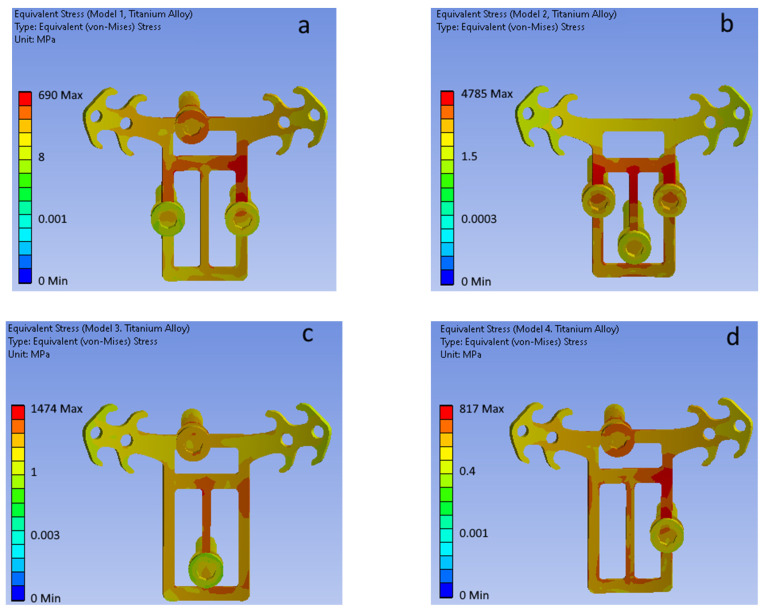
Equivalent stress fields for (**a**–**d**) mini-implant configurations (POD material—titanium alloy). (**a**) Model 1. (**b**) Model 2. (**c**) Model 3. (**d**) Model 4.

Figure 7 shows equivalent (von Mises) stress fields for each of the considered mini-implant configurations (POD is made of titanium alloy).

### 3.2. Stainless Steel 316LS/316LVM

**Figure 8:**
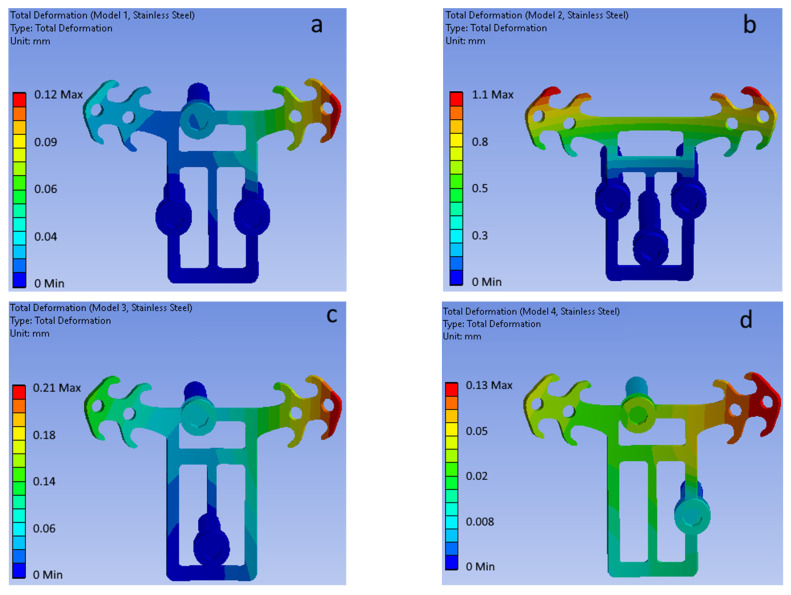
Total displacement fields for (**a**–**d**) mini-implant configurations (POD material—stainless steel). (**a**) Model 1. (**b**) Model 2. (**c**) Model 3. (**d**) Model 4.

Figure 8 shows the total displacement fields of the POD (316LS/316LVM medical stainless-steel material) and mini-implants of the four considered configurations (top view).

**Figure 9:**
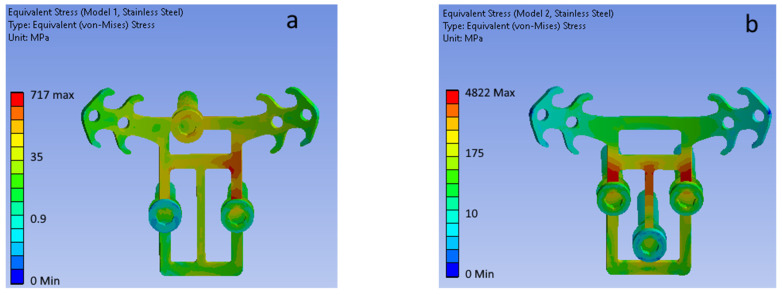

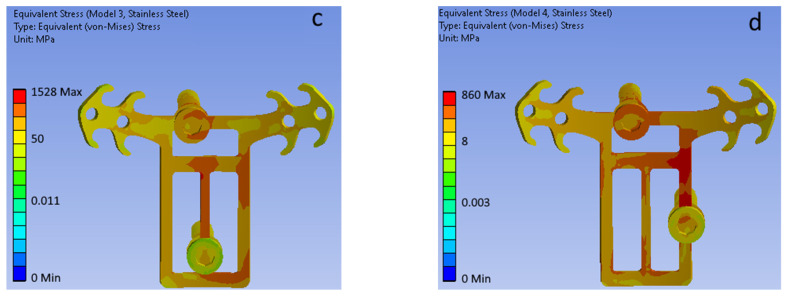
Equivalent stress field for (**a**–**d**) mini-implant configurations (POD material—stainless steel). (**a**) Model 1. (**b**) Model 2. (**c**) Model 3. (**d**) Model 4.

Figure 9 shows equivalent (von Mises) stress fields for each of the considered mini-implant configurations (POD is made of stainless steel).

**Figure 10:**
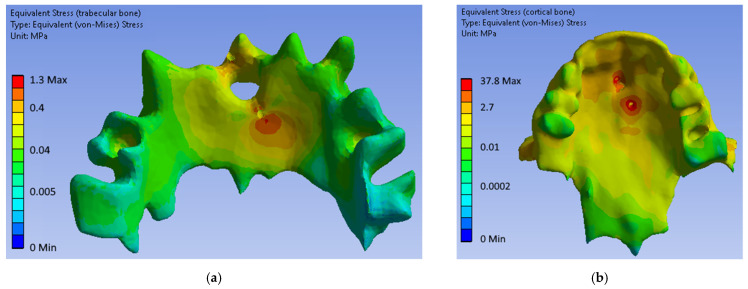
Equivalent stresses in bone tissues for the fourth configuration of mini-implants (Model 4): (**a**) trabecular bone; (**b**) cortical bone.

Figure 10 shows typical equivalent stress fields in bone tissues obtained from numerical simulations. It can be seen that the highest stress values are concentrated in the area of mini-implant installation on the upper threads.

Table 2 summarizes the highest total displacement values of the POD and mini-implants, as well as the highest equivalent stress values for each of the four considered configurations of mini-implants.

Table 3 summarizes maximum cancellous and cortical bone equivalent stresses for each considered mini-implant configuration.

## 4. Discussion

As a result of biomechanical modeling, mechanical stability, and strength of each considered POD and mini-implant, configurations were investigated. The performed biomechanical analysis, as well as the use of biomechanical criteria for assessing the success of surgical treatment [17], made it possible to select one successful (the most stable and durable) mini-implant configuration.

Simulation results showed that the least rigid (least stable) was the second configuration (Table 2) because the front part of POD works like a cantilever beam. For it, the maximal total displacement of POD ranged from 1.20 to 2.30 mm (steel and titanium POD, respectively), which is significantly higher than maximal total displacements for other considered configurations. Therefore, the first and the fourth configurations of mini-implant placement were the most stable.

The second configuration was the least strong, as evidenced by the highest equivalent stresses found in POD (almost 5000 MPa for both the investigated materials). The third configuration also showed low strength, even though in this case the equivalent stresses (1528 and 1475 MPa for stainless steel and titanium alloy, respectively) decreased by more than three times compared to the second. Such stresses indicate that the under-investigated load, POD in these configurations will fail since the ultimate strength of stainless steel and titanium alloy are significantly exceeded. Configurations numbered 1 and 4 were viable under a load of 100 N and give maximum values of equivalent stresses below the yield point of stainless steel and titanium alloy (up to 930 MPa and 790 MPa, respectively) [18,19].

Analysis of the highest equivalent stresses from Table 3 showed that from the biomechanical point of view, the first and fourth configurations were the most preferable. In general, for each considered configuration, trabecular and cortical bone tissues will not be damaged under the considered load, since the ultimate strength has not been reached [17,20].

It should also be noted that the use of steel as POD material increased the rigidity of the structure in comparison with titanium alloy. This is logical since due to the higher modulus of elasticity, the structure becomes more rigid. At the same time, the use of titanium alloy as POD material reduced stresses in it, and, consequently, increased its strength and resistance to the loads considered.

Note that under the considered loads, POD was the most loaded, and mini-implants experienced equivalent stresses not more than 350 MPa, which is permissible even under cyclic loads.

Thus, based on biomechanical criteria for evaluating the success of treatment, it can be concluded that the first and fourth mini-implant configurations are successful and can be recommended for implementation. It should be noted that the second configuration is the least preferable due to the relatively low stability and insufficient strength in comparison with other considered configurations.

The limitation of this work should be attributed to the fact that it investigated POD only under the action of a chewing load. At the same time, it can experience other stresses as well. Our further research will be devoted to this. Despite this, even the considered loading conditions made it possible to determine the variants of mini-implant installation, showing the worst stability and strength from the biomechanical point of view.

## 5. Conclusions

In this work, a comparative biomechanical analysis of various planning options for an individualized design of a palatal anchorage device with a bite plane was carried out. The advantages and disadvantages of each configuration were shown. The most stable and durable construction (the first and the fourth, which provides distributed front and side support of the device) from the biomechanical point of view has been determined. The data obtained are of interest due to the fact that the perception of the chewing load by orthodontic constructions was previously calculated for devices with a fixation on the teeth, and in our work, we have shown the possibility of accepting the load by devices based on orthodontic mini-implants inserted directly into the bone tissue.

## Figures and Tables

**Table 1:** Bone tissues, POD, and mini-implants mechanical properties.

Material Type	Youngs Modulus, MPa	Poisons Coefficient
Trabecular bone	1000	0.3
Cortical bone	13,800	0.3
Stainless steel 316LS/316LVM	195,000	0.3
Titanium alloy Ti6Al4V ELI	110,000	0.35

**Table 2:** Total displacement, equivalent stress highest values in POD, and mini-implants.

	POD Material	Model 1	Model 2	Model 3	Model 4
Max total displacement, mm	Steel	0.12	1.10	0.21	0.13
	Titanium alloy	0.18	2.30	0.34	0.21
Max Equivalent stress, MPa	Steel	717	4822	1528	860
	Titanium alloy	666	4785	1475	717

**Table 3:** Equivalent stress highest values in trabecular and cortical bone.

Max Equivalent Stress, MPa	POD Material	Model 1	Model 2	Model 3	Model 4
Trabecular bone	Steel	1.7	3.5	0.9	1.3
	Titanium alloy	1.5	3.2	1.1	1.6
Cortical bone	Steel	32.4	78.7	77.6	37.8
	Titanium alloy	38.7	102.2	118.4	53.4

## Data Availability

All data generated or analyzed during this study are available from authors with a request.
